# Upward-directed exit-site of the swan-neck catheter and “Easy-to-disinfect the backside area of exit-site” may prevent PD complications

**DOI:** 10.1007/s10157-023-02454-7

**Published:** 2024-02-10

**Authors:** Kyohei Ogawa, Masato Ikeda, Izumi Shirai, Kentaro Ohshiro, Yukio Maruyama, Takashi Yokoo, Yudo Tanno, Hiroyuki Terawaki, Tsutomu Sakurada, Kazuhiro Yoshikawa, Hironobu Inoue, Chieko Higuchi, Tomohiro Kaneko, Fumiaki Nogaki, Atsushi Ueda, Yoshitaka Maeda

**Affiliations:** 1 Division of Nephrology and Hypertension, The Jikei University Kashiwa Hospital, 163-1 Kashiwashita, Kashiwa, Chiba 277-8567 Japan; 2 Division of Nephrology and Hypertension, The Jikei University School of Medicine, Tokyo, Japan; 3 Division of Nephrology and Hypertension, Katsushika Medical Center, Tokyo, Japan; 4 Division of Nephrology, Department of Internal Medicine, Teikyo University Chiba Medical Center, Chiba, Japan; 5 Division of Nephrology and Hypertension, Department of Internal Medicine, Saint Marianna University School of Medicine, Kanagawa, Japan; 6 Department of Nephrology and Rheumatology, Iwate Prefectural Central Hospital, Iwate, Japan; 7 Department of Nephrology, Saiseikai Kumamoto Hospital, Kumamoto, Japan; 8 Department of Nephrology, Tokyo Women’s Medical University, East Medical Hospital, Tokyo, Japan; 9 Department of Nephrology, Nippon Medical School Tamanagayama Hospital, Tokyo, Japan; 10 Department of Nephrology, Shimada Municipal Hospital, Shizuoka, Japan; 11 Department of Nephrology, Hitachi General Hospital, Ibaraki, Japan; 12 Nephrology Division, Department of Internal Medicine, JA Toride Medical Center, Toride, Japan

**Keywords:** Peritoneal dialysis, Exit-site, Direction, Infection, Catheter, Swan-neck, Easy-to-see, Disinfect, Dislocation, Peritonitis

## Abstract

**Background:**

Upward-directed exit-site has been believed to be the worst for frequent ESI by an old retrospective study using straight catheters. No comparison study of 3 exit-site directions using swan-neck catheter has been performed regarding which direction is the best for our endpoints, Easy-to-see the backside area of exit-site: ESBE, Easy-to-disinfect the backside area of exit-site: EDBE, reduction of both exit-site infection (ESI), symptomatic catheter dislocation and peritonitis.

**Methods:**

We assessed the relationship of exit-site direction with our endpoints in a quantitative cross-sectional, multicentered questionnaire survey. Patients who received either non-surgical catheter implantation or exit-site surgery were excluded.

**Results:**

The numbers (percentage) of exit-site directions in included 291 patients were upward 79 (26.0), lateralward 108 (37.5) and downward 105 (36.5). Cochran-Armitage analysis showed a significant step-ladder increase in the prevalence of ESI as the direction changed from upward to lateralward to downward (0.15 ± 0.41, 0.25 ± 0.54, 0.38 ± 0.69 episodes/patient-year, *p* = 0.03). Multivariable regression analysis revealed the upward exit-site independently associates with both higher frequency of ESBE (OR 5.55, 95% CI 2.23–16.45, *p* < 0.01) and reduction of prevalence of ESI (OR 0.55, 95%CI 0.27–0.98, *p* = 0.04). Positive association between the prevalence of symptomatic catheter dislocation and ESI (OR 2.84, 95% CI 1.27–7.82, *p* = 0.01), and inverse association between EDBE and either prevalence of symptomatic catheter dislocation (OR 0.27, 95% CI 0.11–0.72) or peritonitis (OR 0.48, 95% CI 0.23–0.99) observed.

**Conclusion:**

Upward-directed swan-neck catheter exit-site may be the best for both ESBE and prevention of ESI. EDBE may reduce catheter dislocation and peritonitis. Symptomatic catheter dislocation may predict ESI.

## Introduction

PD catheter implantation techniques are thought to be essential to reduce catheter-related complications and the ideal exit-site direction which helps daily care and reduce PD complications should be updated [1].

Historically, Twardowski et al. first compared all 4 tunnel directions (not exit-site directions) by using only straight catheters [2] and found the upward tunnel direction left a worse outcome of ESI without significant difference. After that, many doctors are implanting catheter exit-site facing a downward direction without crucial evidence [3, 4]. Additionally, they proposed a non-straight, new swan-neck tunnel catheter.

Following this proportion, Crabtree et al. reported no significant difference between the lateralward straight catheters and the downward swan neck catheters about prevalence rates of ESI and tunnel infections, peritonitis, and catheter loss [5]. In response to this report, ISPD recommends a lateralward or downward direction of PD catheter [6].

**Fig. 1:**
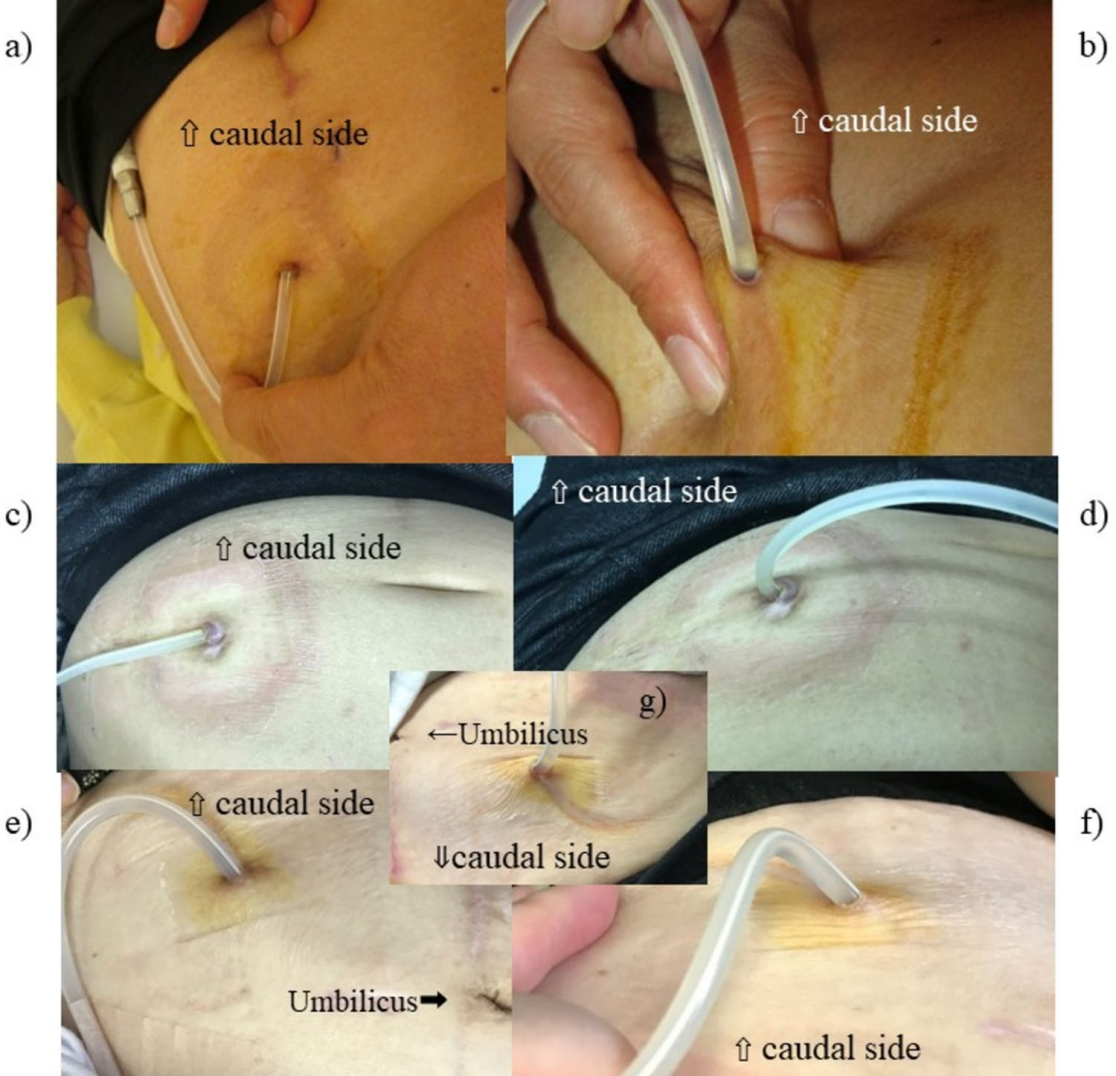
Representative photos of the 3 directions of the catheter exit-site. **a**–**f** Each photo was taken from the patient’s cranial side to show the patient’s view. The 3 catheters’ natural state (**a**, **c**, **e**) and holding up state (**b**, **d**, **f**) (to see the hidden back side of the catheter) are shown separately. **a**, **b** Upward-directed catheter exit-site placed at left UAE. **c**, **d** Lateralward-directed catheter exit-site at left UAE. **e**, **f** Downward-directed catheter exit-site at left LAE. **g** A photo of exit-site which was taken from doctor’s view showing the hidden back side of the catheter by holding up the catheter. Exit-site was viewed by physician on a sitting position, in front of the patient who is also on a sitting position while they are face to face. Doctors could see the hidden backside area of catheter clearly from their view, but this patient could not see the area fully and had to use a mirror to see her catheter

We have experienced several patients complaining they have to pull and bend their catheter toward an upward position to see and disinfect the backside area of exit-site. Usually, the majority of the PD patients can easily see the surface area of all the 3 exit-site directions. On the other hand, the backside area of the exit-site seemed frequently hard to see for the patients who have downward-directed directed exit-site. It is because their catheter itself is blocking their view toward the backside area of the catheter, particularly in the case of patients having a downward-directed catheter (Fig. 1). If the backside area is not disinfected appropriately every day, the backside area would become susceptible to causative organism because of its enclosed space nature.

Further, the mean age of Japanese patients who initiate PD has reached over 70 years old and their presbyopia, weak grasping power and poor concentration might impair ESBE and/or EDBE.

If patients cannot see the backside area of the catheter exit-site fully, they may pull or bend their catheter to see the backside area of exit-site more directly. This frequent mechanical stress on exit-site may lead to not only ESI [7] but also symptomatic catheter dislocation [8].

Currently, we can produce 3 natural directions of catheter [9] by using 2 types of swan neck catheters. However, there has been no report examining the 3 exit-site facing directions altogether using only swan-neck catheters [1–5].

So, we hypothesized patients having upward-directed swan-neck catheter exit-site may feel ESBE and EDBE compared to patients having downward or lateral exit-site, which finally associate with reduction of the prevalence of ESI, peritonitis and symptomatic catheter dislocation.

Our primary endpoints were the prevalence of ESI, secondary endpoints were ESBE, EDBE, the prevalence of either peritonitis or symptomatic catheter dislocation.

This study's objective was to investigate whether the upward-directed swan-neck catheter exit-site associates with ESBE, EDBE, the prevalence of ESI, peritonitis, and symptomatic catheter dislocation or not. The best catheter direction for the elderly should be also re-examined [10].

## Methods

A quantitative cross-sectional, multicentered questionnaire survey was performed at 12 institutions which belong to Japanese Society of Interventional Nephrology [11] in accordance to the ethics committee for clinical research at Jikei University School of Medicine approved all protocols in this study [Permission no. 28–269 (8512)].

First, the questionnaire was distributed to the patients who have been implanted with a swan-neck catheter, could answer by themselves, and who were already undergoing PD treatment for 6 or more months at the time of answering the questionnaire between January 2017 and July 2022. Second, the attending physician confirmed the accuracy of the patients’ answers and corrected the mistakes as appropriate. The correct information was obtained not only by medical records but also by both direct questions to patients and direct observation of the exit-site from each outpatient physical examination.

In greater detail, we created a question “Do you think the backside area of your exit-site is easy-to-see?” And each patient selected one of the answers as Yes, No or Not Sure. If the patients answered Yes, it was defined as “easy-to-see the backside area of exit-site: ESBE”.

And we also created another question “Do you think the backside area of your exit-site is easy-to-disinfect?” If the patients answered Yes, it was defined as “easy-to-disinfect the backside area of exit-site: EDBE”.

The direction of PD catheter was divided into 3 categories: upward, lateralward, and downward direction (Fig. 1). Attending physicians answered catheter direction as lateralward if the catheter at exit-site comes out of the subcutaneous tunnel in a horizontal direction showing right and left side. If the catheter at the exit-site comes out of the subcutaneous tunnel in slightly off the lateral side, physicians selected upward or downward as appropriate. We used the final direction of the catheter exit-site, which was after 6 or more months of catheter usage at the time of survey. Both the upper abdominal exit-site (UAE) and the lower abdominal exit-site (LAE) were defined as catheters located at the upper or lower area of the umbilicus. A symptomatic catheter dislocation was defined by an episode of flow dysfunction with catheter deviation from pelvic space to the other space diagnosed by X-ray or computed tomography.

All attending nephrologists are Japanese certified dialysis specialists and they used ISPD guidelines to diagnose ESI [6] and peritonitis [12] as appropriate and they did not use any topical prophylactic antibiotic protocols because the Japanese insurance system does not allow them to use them at the PD catheter exit-site. All nephrologists and nurses taught patients exit-site care with a disinfectant solution every day.

### Inclusion and exclusion criteria

395 PD patients belonging to 12 institutions who were undergoing PD treatment without HD concomitant for 6 or more months were screened. To reduce institutional bias, only institutions surgically implanting 2 or more directions of PD catheter by nephrologists [11] were included. We excluded incontinence and blind patients who could not see the exit-site and included only those who could disinfect by themselves. By the inclusion and exclusion criteria, 1 institution (20 patients) which implants PD catheter by Seldinger technique (non-surgical procedure) and 3 institutions (59 patients) with only a single direction of the catheter (such as the institutions that always implanted only downward-directed catheter) were excluded. Further, we excluded 25 patients who might change their exit-site direction by surgical procedure on the exit-site and analyzed the resultant 291 patients.

### Application of catheters in this study

Japanese catheter manufacturer, Hayashidera Co., Ltd., (Kanazawa, Japan) supplies 2 different angle catheters, one is an inverted U-shaped catheter for downward direction (JB-5, JB-6 (A), SP-1) and the other is a 50 degrees angle swan-neck catheter designed for both diagonally upward and lateral ward direction (JB1-130, JB2-130, JB4-130).

The standard decision procedures for both the exit-site location and the catheter type in the finally included 8 institutions were as follows: first, each exit-site location was decided by the patient’s wishes, hair place, surgical wound, and belt line. Next, the catheter insertion site and deep cuff location were selected by each nephrologist to achieve the proper pelvic position of a straight catheter tip. Third, the nephrologists selected the best catheter type and length to fit these conditions among the catheters that each institution adopts. Finally, the direction of the catheter was naturally formed without any bending procedure.

### Statistics

Statistical analyses were performed using JMP 9.0 (SAS Institute Inc., Cary, NC, USA). Data are expressed as the mean ± standard deviation or numbers (percentage) of patients. Comparisons across the various groups were performed using the Pearson Chi-square test for categorical data, the Dunnett test for continuous data, and ANOVA for tertile analysis. All tests were two-tailed, and a *p* value of < 0.05 was considered significant. The association of 3 catheter directions and prevalence of ESI, ESBE, and EDBE were analyzed by Cochran–Armitage analysis. Associated factors on univariate analysis were subsequently included in a multivariate model. *T* values and 95% confidence intervals (CIs) were determined using univariate and multivariate logistic regression models for the factors that were significantly associated with outcomes.

## Results

We finally included 291 PD patients at 8 institutions and their mean PD duration was 3.3 ± 2.9 years. Among these, 19 patients utilized SMAP (Stepwise initiation of peritoneal dialysis using Moncrief and Popovich technique) and no patient used SPIED (Short-term PD Induction and Education technique).

### Comparison of patient characteristics among 3 directions of catheter exit-sites

Table 1 shows the characteristic comparison of 3 categorized groups divided by 3 catheter exit-site directions. The numbers (percentage) of exit-site directions were upward 79 (25.8), lateralward 108 (37.1), and downward 108 (37.1). Mean prevalence rates of ESI, peritonitis, and symptomatic catheter dislocation were 0.27 ± 0.58, 0.24 ± 0.59, and 0.06 ± 0.31 episodes/patient-year, respectively. These mean prevalence rates were used to further compare higher and lower frequency groups’ characteristics.

**Table 1:** Comparison of patient characteristics among 3 directions of catheter exit-sites

	Overall	Upward	Lateralward	Downward	*p* value
Subject *n*, (%)	291 (100%)	79 (25.8)	108 (37.1)	108 (37.1)	
Age (mean ± SD)	63.1 ± 13.3	61.3 ± 14.6	62.2 ± 13.5	65.3 ± 11.9	0.08
Male *n*, (%)	198 (68.0)	51 (68.0)	79 (73.2)	68 (63.0)	0.28
Diabetic kidney disease *n*, (%)	128 (44.0)	33 (44.0)	44 (40.7)	51 (47.2)	0.63
PD duration (year)	3.3 ± 2.9	3.1 ± 2.6	3.3 ± 2.9	3.5 ± 3.3	0.65
ESI prevalence (episodes/patient-year)	0.27 ± 0.58	0.15 ± 0.41	0.25 ± 0.54	0.38 ± 0.69	0.03
ESI prevalence > 0.27 *n*, (%)	65 (22.3)	10 (13.3)	23 (21.3)	32 (29.6)	0.03
Peritonitis prevalence (episodes/patient-year)	0.24 ± 0.59	0.18 ± 0.44	0.29 ± 0.77	0.23 ± 0.44	0.46
Peritonitis prevalence > 0.24 *n*, (%)	69 (23.7)	15 (20.0)	27 (25.0)	27 (25.0)	0.68
Symptomatic catheter dislocation prevalence (episodes/patient-year)	0.06 ± 0.31	0.01 ± 0.09	0.08 ± 0.40	0.08 ± 0.30	0.27
Dislocation prevalence > 0.06 (episodes/patient-year) *n*, (%)	24 (8.2)	3 (4.0)	9 (8.3)	12 (11.1)	0.23
UAE *n*, (%)	166 (57.0)	68 (90.7)	65 (60.2)	33 (30.6)	< 0.01
LAE *n*, (%)	125 (43.0)	7 (9.3)	43 (39.8)	75 (69.4)	< 0.01
ESBE *n*, (%)	218 (74.9)	69 (92.0)	80 (74.1)	69 (63.9)	< 0.01
EDBE *n*, (%)	250 (85.9)	68 (90.7)	95 (88.0)	87 (80.6)	0.04

A significant step-ladder decrease in the prevalence of ESI (episodes/patient-year) was noted as the catheter direction changed from downward to lateral to upward (downward: 0.38 ± 0.69, lateralward: 0.25 ± 0.54, upward: 0.15 ± 0.41, *p* = 0.03).

A significant step-ladder increase in frequency greater than of mean prevalence of ESI (0.27 episodes/patient-year) was noted as the catheter direction changed from upward to lateralward to downward (upward: 13.3%, lateralward: 21.3%, downward: 29.6%, *p* = 0.03).

A non-significant step-ladder decrease in frequency greater than of mean prevalence of symptomatic dislocation prevalence (> 0.06 episodes/patient-year) was seen as the catheter direction changed from downward to lateral to downward (11.1, 8.3, 4.0%, *p* = 0.09).

On the other hand, there was no step-ladder change in the prevalence of peritonitis by exit-site direction.

Cochran-Armitage analysis revealed a significant step-ladder decrease in the frequency of ESBE as the catheter direction changed from upward to lateralward to downward (92.0, 74.1, 63.9%,* p* < 0.01, respectively. The frequency of EDBE also showed a significant step-ladder decrease as the catheter direction changed from upward to lateralward to downward (90.7, 88.0, and 80.6%, *p* = 0.04).

As shown in Table 2, multivariable regression analysis using the upward-directed catheter as an objective variable revealed the upward-directed catheter independently associated with ESBE (OR 5.55, 95% CI 2.23–16.45, *p* < 0.01) and the prevalence of ESI (episodes/patient-year) (OR 0.55, 95% CI 0.27–0.98, *p* = 0.04).

**Table 2:** Univariable and multivariable logistic regression models to identify covariates associated with the upward-directed catheter

	Univariable model		Multivariable model	
	OR (95% CI)	*p* value	OR (95% CI)	*p* value
ESBE *n*, (%)	5.17 (2.30–13.86)	< 0.01	5.55 (2.23–16.45)	< 0.01
EDBE *n*, (%)	1.81 (0.81–4.64)	0.15	1.31 (0.46–3.58)	0.60
ESI prevalence (episodes/patient-year)	0.52 (0.26–0.91)	0.02	0.55 (0.27–0.98)	0.04

### Comparison of characteristics between UAE and LAE

Next, we compared patient characteristics between UAE and LAE. The numbers (percentage) of exit-site positions were UAE 166 (57.0%) and LAE 125 (43.0%).

The UAE group showed a significantly higher frequency of ESBE compared to the LAE group (81.3% vs. 66.4%, *p* < 0.01). And worse ESI prevalence rate was observed in the UAE group compared to the LAE group (0.32 ± 0.64 vs. 0.21 ± 0.49, *p* = 0.10). However, there was no significant difference in the prevalence of ESI, peritonitis, symptomatic catheter dislocation, and frequency of EDBE between 2 groups. (Table 3).

**Table 3:** Comparison of characteristics between UAE and LAE

	UAE	LAE	*p* value
Subject *n*, (%)	166 (57.0)	125 (43.0)	
Age (mean ± SD)	62.1 ± 13.8	64.4 ± 12.5	0.15
Male *n*, (%)	51 (30.7)	42 (22.6)	0.60
Diabetic kidney disease *n*, (%)	75 (45.2)	53 (42.4)	0.64
PD duration (year)	3.2 ± 2.5	3.6 ± 3.5	0.47
Peritonitis prevalence (episodes/patient-year)	0.22 ± 0.48	0.27 ± 0.71	0.51
ESI prevalence (episodes/patient-year)	0.32 ± 0.64	0.21 ± 0.49	0.10
Symptomatic catheter dislocation prevalence (episodes/patient-year)	0.06 ± 0.32	0.07 ± 0.29	0.61
ESBE *n*, (%)	135 (81.3)	83 (66.4)	< 0.01
EDBE *n*, (%)	146 (88.0)	104 (83.2)	0.25

### Comparison of patient characteristics between higher and lower ESI prevalence group

The group with higher ESI prevalence > 0.27 (episodes/patient-year) showed a significantly higher frequency of both downward-directed catheter (49.2% vs 33.6%, *p* = 0.02) and symptomatic catheter dislocation prevalence (episodes/patient-year) (0.17 ± 0.54 vs 0.03 ± 0.19, *p* < 0.01) compared to the group with lower ESI prevalence ≤ 0.27 (episodes/patient-year). And the higher ESI group also showed a significantly lower frequency of upward-directed catheters (15.4% vs 28.8%, *p* = 0.03) compared to the lower ESI group (Table 4). Neither ESBE nor EDBE is associated with the prevalence of ESI significantly.

**Table 4:** Comparison of characteristics between patients with ESI prevalence ≤ 0.27 and > 0.27 episodes/patient-year

	ESI prevalence ≤ 0.27 episodes/patient-year	ESI prevalence > 0.27 episodes/patient-year	*p* value
Subject *n*, (%)	235 (74.4)	81 (25.6)	
ESI prevalence (episodes/patient-year)	0.02 ± 0.05	1.16 ± 0.69	< 0.01
Age (mean ± SD)	63.6 ± 13.3	61.3 ± 13.4	0.22
Male *n*, (%)	160 (70.8)	38 (58.5)	0.06
Diabetic kidney disease *n*, (%)	97 (42.9)	31 (47.7)	0.49
PD duration (year)	3.4 ± 3.1	3.1 ± 2.6	0.44
Peritonitis prevalence (episodes/patient-year)	0.24 ± 0.62	0.25 ± 0.47	0.93
Symptomatic catheter dislocation prevalence (episodes/patient-year)	0.03 ± 0.19	0.17 ± 0.54	< 0.01
Catheter directions			
Upward *n*, (%)	65 (28.8)	10 (15.4)	0.03
Lateralward *n*, (%)	85 (37.6)	23 (35.4)	0.74
Downward *n*, (%)	76 (33.6)	32 (49.2)	0.02
ESBE *n*, (%)	173 (76.6)	45 (69.2)	0.23
EDBE *n*, (%)	196 (86.7)	54 (83.1)	0.46

As shown in Table 5), multivariate logistic regression analysis revealed the higher ESI group was independently associated with both the prevalence of symptomatic catheter dislocation (episodes/patient-year) (OR 2.84, 95% CI 1.27–7.82, *p* = 0.01) and the upward-directed catheter (OR 0.49, 95% CI 0.22–0.99, *p* = 0.045) inversely.

**Table 5:** Univariable and multivariable logistic regression models to identify covariates associated with ESI prevalence > 0.27 episodes/patient-year

	Univariable model		Multivariable model	
	OR (95% CI)	*p* value	OR (95% CI)	*p* value
Upward-directed catheter	0.45 (0.21–0.90)	0.02	0.49 (0.22–0.99)	0.045
Catheter dislocation prevalence (episodes/patient-year)	3.10 (1.38–8.53)	< 0.01	2.84 (1.27–7.82)	0.01

### Comparison of patient characteristics between higher and lower symptomatic catheter dislocation prevalence group

The group with a higher prevalence of symptomatic catheter dislocation (> 0.06 episodes/patient-year) showed significantly longer PD duration (4.9 ± 4.5 vs 3.2 ± 2.8 years, *p* < 0.01) and higher frequency of EDBE (87.6% vs. 66.7%, *p* < 0.01) compared to the group with lower prevalence of symptomatic catheter dislocation (≤ 0.06 episodes/patient-year).

The higher dislocation group showed a higher prevalence rate of ESI (0.49 ± 0.85 vs 0.25 ± 0.55 episodes/patient-year, *p* = 0.05), but did not reach statistical significance (Table 6). Cochran-Armitage analysis showed a step-ladder increase in the frequency of higher dislocation (> 0.06 episodes/patient-year) as the exit-site direction change from upward to lateral, downward direction, but it did not reach statistical significance (12.5, 37.5, 50.0, *p* = 0.09).

**Table 6:** Comparison of characteristics between patients with symptomatic catheter dislocation prevalence ≤ 0.06 and > 0.06 episodes/patient-year

	Symptomatic catheter dislocation prevalence ≤ 0.06 (episodes/patient-year)	> 0.06 (episodes/patient-year)	*p value*
Subject *n*, (%)	267 (91.8)	24 (8.2)	
Age (mean ± SD)	63.5 ± 13.1	58.1 ± 14.4	0.06
Male *n*, (%)	179 (67.0)	19 (79.2)	0.22
Diabetic kidney disease *n*, (%)	118 (44.2)	10 (41.8)	0.81
PD duration (year)	3.2 ± 2.8	4.9 ± 4.5	< 0.01
Peritonitis prevalence (episodes/patient-year)	0.25 ± 0.60	0.19 ± 0.45	0.66
Positive episode of ESI *n*, (%)	75 (28.1)	12 (50.0)	0.03
ESI prevalence (episodes/patient-year)	0.25 ± 0.55	0.49 ± 0.85	0.05
ESI prevalence > 0.27 *n*, (%)	57 (21.4)	8 (33.3)	0.18
Catheter direction			
Upward *n*, (%)	72 (27.0)	3 (12.5)	0.12
Lateralward *n*, (%)	99 (37.1)	9 (37.5)	0.97
Downward *n*, (%)	96 (36.0)	12 (50.0)	0.17
ESBE *n*, (%)	201 (75.3)	17 (70.8)	0.63
EDBE *n*, (%)	234 (87.6)	16 (66.7)	< 0.01
UAE *n*, (%)	155 (58.1)	11 (45.8)	0.25

Multivariate logistic regression analysis revealed an independent association between prevalence rates of symptomatic catheter dislocation and EDBE (OR 0.27, 95% CI 0.11–0.72. *p* = 0.01) with the presence of confounding factor, PD duration (year) (OR 1.16, 95% CI 1.03–1.29, *p* = 0.02) (Table 7).

**Table 7:** Univariable and multivariable logistic regression models to identify covariates associated with higher prevalence rates of symptomatic catheter dislocation (> 0.06 episodes/patient-year)

	Univariable model		Multivariable model	
	OR (95% CI)	*p* value	OR (95% CI)	*p* value
PD duration (year)	1.15 (1.03–1.28)	0.02	1.16 (1.03–1.29)	0.02
EDBE	0.28 (0.11–0.74)	0.01	0.27 (0.11–0.72)	0.01

### Comparison of patient characteristics between higher and lower peritonitis prevalence group

The group with higher prevalence of peritonitis (> 0.24 episodes/patient-year) showed significantly lower frequency of EDBE (78.3 vs 88.3 episodes/patient-year, *p* = 0.04) and shorter PD duration (2.5 ± 1.8 vs 3.6 ± 3.2 years, *p* < 0.01) compared to the group with lower prevalence of peritonitis (≤ 0.24 episodes/patient-year) (Table 8). There were no significant differences in the exit-site directions between the 2 groups.

**Table 8:** Comparison of characteristics between patients with peritonitis prevalence ≦0.24 and > 0.24 episodes/patient-year

	Peritonitis prevalence ≤ 0.24 episodes/patient-year	Peritonitis prevalence > 0.24 episodes/patient-year	*p* value
Subject *n*, (%)	222 (76.3)	69 (23.7)	
Age (mean ± SD)	62.4 ± 12.8	65.3 ± 14.6	0.12
Male *n*, (%)	149 (67.1)	49 (71.0)	0.54
Diabetic kidney disease *n*, (%)	100 (45.1)	28 (40.6)	0.51
PD duration (year)	3.6 ± 3.2	2.5 ± 1.8	< 0.01
ESI prevalence (episodes/patient-year)	0.28 ± 0.61	0.24 ± 0.49	0.62
Positive episode of dislocation *n*, (%)	19 (8.6)	6 (8.7)	0.97
Symptomatic catheter dislocation prevalence (episodes/patient-year)	0.06 ± 0.32	0.06 ± 0.27	0.87
Catheter directions			
Upward *n*, (%)	60 (27.0)	15(21.7)	0.38
Lateralward *n*, (%)	81 (36.5)	27 (39.1)	0.69
Downward *n*, (%)	81 (36.5)	27 (39.1)	0.69
UAE *n*, (%)	128 (57.7)	38 (55.1)	0.70
ESBE *n*, (%)	168 (75.7)	50 (72.5)	0.59
EDBE *n*, (%)	196 (88.3)	54 (78.3)	0.04

Multivariable logistic regression analysis revealed independent association between the prevalence of peritonitis and EDBE (OR 0.48, 95% CI 0.23–0.99. *p* = 0.047) with the presence of cofounding factor, PD duration (year) (OR 0.86, 95% CI 0.75–0.96, *p* < 0.01) (Table 9).

**Table 9:** Univariable and multivariable logistic regression models to identify covariates associated with peritonitis prevalence > 0.24 episodes/patient-year

	Univariable model		Multivariable model	
	OR (95%CI)	*p* value	OR (95%CI)	*p* value
PD duration (year)	0.86 (0.75–0.96)	< 0.01	0.86 (0.75–0.96)	< 0.01
EDBE	0.48 (0.24–0.98)	0.04	0.48 (0.23–0.99)	0.047

### Comparison of characteristics between patients aged ≤ 55 and > 55

Accommodation function in the human eye declines with age in the development of presbyopia and most people experience a near-total loss of accommodative ability by 55 years old [13, 14].  So, we compared our participants aged ≤ 55 and > 55. Our patients older than 55 years of age significantly felt ESBE less frequently compared to patients aged 55 or under (*n* = 147/206, 71.4% vs. *n* = 71/85, 83.5%, *p* = 0.03). They also felt EDBE less frequently compared to patients aged 55 or under (n = 173/206, 84.0% vs. 77/85, 90.6%, *p* = 0.14), but did not reach statistical significance (Table 10). There were no significant differences in the exit-site directions between the 2 groups. And there were no significant differences in the prevalence rates of ESI, peritonitis, symptomatic dislocation between the patients older than 55 and 55 years old or younger.

**Table 10:** Comparison of characteristics between patients aged ≤ 55 and > 55

	Age ≤ 55	Age > 55	*p* value
Subject *n*, (%)	85 (29.2)	206 (70.8)	
Age (mean ± SD)	46.8 ± 6.8	60.8 ± 8.7	< 0.01
Male *n*, (%)	53 (62.4)	145 (70.4)	0.18
Diabetic kidney disease *n*, (%)	42 (49.4)	86 (41.8)	0.23
PD duration (year)	3.1 ± 3.1	3.4 ± 2.9	0.47
Peritonitis prevalence (episodes/patient-year)	0.18 ± 0.42	0.27 ± 0.64	0.27
ESI prevalence (episodes/patient-year)	0.28 ± 0.61	0.27 ± 0.57	0.94
Symptomatic catheter dislocation prevalence (episodes/patient-year)	0.07 ± 0.33	0.06 ± 0.30	0.69
Catheter directions			
Upward *n*, (%)	24 (28.2)	51 (24.8)	0.54
Lateralward *n*, (%)	36 (42.4)	72 (35.0)	0.23
Downward *n*, (%)	25 (29.4)	83 (40.3)	0.08
ESBE *n*, (%)	71 (83.5)	147 (71.4)	0.03
EDBE *n*, (%)	77 (90.6)	173 (84.0)	0.14
UAE *n*, (%)	55 (64.7)	111 (53.9)	0.09

## Discussion

We first examined the 3 directions of swan-neck catheter exit-site altogether for their association with ESBE, EDBE and the prevalence of 3 complications for PD; ESI, symptomatic catheter dislocation, and peritonitis by a quantitative cross-sectional, multicentered questionnaire survey. The prevalence rates of ESI and peritonitis in our participants (0.27 and 0.24 episodes/patient-year) were similar to the Japanese standard prevalence (0.35 and 0.22 episodes/patient-year) [15]. Surprisingly, this study was the first to suggest the upward-directed swan-neck catheter exit-site is the best, but the downward-directed swan-neck catheter exit-site is the worst for both ESBE, EDBE, and prevention of ESI among all 3 directions. Interestingly, non-significantly worse ESI prevalence was observed in the UAE group compared to the LAE group (0.32 ± 0.64 vs. 0.21 ± 0.49, *p* = 0.10) in spite of the significantly higher frequency of ESBE in the UAE group, suggesting the upward direction of exit-site may be more effective to prevent ESI rather than the upper abdominal exit-site position.

Currently, ISPD recommends lateral or downward direction of PD catheter [6] by main reference of Crabtree JH et al. [5], which compared only 2 directions, lateral straight catheter, and downward swan-neck catheter, and did not examine the upward exit-site. On the other hand, our patients showed significant differences in ESI prevalence among downward, lateral, and upward swan-neck catheter (0.38 vs 0.25 vs 0.15 episodes/patient-year, *p* = 0.03, respectively).

Several reasons seemed to be possible which caused different results in regard to the prevalence rates of ESI between this study and Crabtree’s report [5].

Firstly, a great difference in the follow-up period was observed between the 2 studies. Our participants continued PD treatment 1.4 years longer than theirs (1.9 vs 3.3 years), so it might contribute to the effective detection of the prevalence of ESI.

Secondly, different types of catheters (straight vs. swan-neck catheter) are candidate causes. Crabtree JH et al. implanted a straight catheter by physically bending it to create lateral exit sites [5], different from the currently used swan-neck catheter in our institutions which naturally produce both upward and lateral catheters as described in the Method.

Thirdly, the mean age difference was noted between ours; 63.1, and theirs; 54.1 years old. Most people experience presbyopia by 55 years old [13, 14] and our patients older than 55 years of age significantly felt ESBE less frequently compared to patients aged 55 or under (*n* = 147/206, 71.4% vs. *n* = 71/85, 83.5%, *p* = 0.03), suggesting presbyopia may take a part of the cause for the lack of ESBE.

Frailty in elderly patients also might influence the non-significantly lower frequency of EDBE in the elderly group (84.0 vs 90.6%, *p* = 0.14). However, we do not have any information about both the visual changes and frailty to demonstrate these specific reasons.

ESBE was independently associated with upward direction of catheter exit-site (OR 5.77, 95% CI 2.35–16.96, *p* < 0.01) and EDBE (OR 14.14, 95% CI 6.46–33.65, *p* < 0.01). From the perspective of PD patients, ESBE is helpful for daily care and may lead to the prevention of ESI. Indeed, the ESBE positive group showed a relatively lower prevalence of ESI compared to the ESBI negative group (0.40 ± 0.69 vs 0.24 ± 0.53, *p* = 0.09), though the difference was not statistically significant. This result suggested that ESBE is not enough to prevent ESI by itself alone. Even if the patients having an upward exit-site could see the backside area of the exit-site fully, all of these patients would not always perform sufficient daily care for their exit site to prevent ESI. Without consistent proper daily care, full ESI prevention would not be achieved. The prevention of ESI will not be guaranteed even if the patients have upward directed exit-site if they do not see or disinfect the exit-site properly and consistently.

To our knowledge, the present study first reported the prevalence of symptomatic catheter dislocation independently associated with EDBE inversely even after adjusting by PD duration. Jianxiong Lin, et al. [8] reported a history of catheter-pulling injury and mechanical stress on the catheter were risk factors for ESI. Indeed, a patient with any difficulty in disinfecting exit-site may have frequent opportunities to give mechanical stress on the catheter exit-site by pulling or bending the catheter frequently to disinfect exit site fully.

Though prevalence rates of catheter dislocation have not been fully elucidated to date, Io et al. reported 12/16 cases of developed catheter dislocation [16]. It accounts for over 31.1 episodes/patient-year, greatly different from our participants (0.06 episodes/patient-year). This may be because they included asymptomatic patients who developed slight dislocation from the Douglas pouch, unlike our participants who developed symptomatic effluent failure by catheter dislocation, suggesting majority of patients who having catheter dislocation may develop no symptom.

In conclusion, this study firstly compared the 3 directions of the swan-neck catheter exit-site altogether and suggested the upward-directed swan-neck catheter exit-site is the best, but the downward-directed swan-neck catheter exit-site is the worst for both ESBE and prevention of ESI among all 3 directions. We may propose to break away from the convention of not using upward directed catheter exit site.

## Limitations

This study is limited by its cross-sectional design and the study size was not enough to demonstrate the superiority of the upward-directed exit-site. There was an asymmetric distribution of an upward exit-site group of patients at each institution for the nature of this non-randomized study. No uniformity among facilities in the management and care of catheter exit-site influenced the results. We do not have details of each patient’s PD schedule involving automated PD. And we could not exclude any possibilities of slight changes in the exit-site direction during the observation period.

Lower prevalence rate of symptomatic catheter dislocation in our participants also should be noted.

The study entry period was long, but the used catheter and surgical implantation procedure did not changed during the study period in the included 8 institutions.

However, we believe this study may give the opportunity to re-investigate the clinical significance of upward-directed swan-neck catheter exit-site. Additional research is necessary to confirm the superiority of upward-directed swan-neck catheters.

## Data Availability

The datasets generated and analyzed during the current study are not publicly available due to containing additional unpublished data but are available from the corresponding author on reasonable request.

## Abbreviations

PD: Peritoneal dialysis
ESI: Exit-site infection
ESBE: Easy-to-see the backside area of exit-site
EDBE: Easy-to-disinfect the backside area of exit-site
UAE: Upper abdominal exit-site
LAE: Lower abdominal exit-site
ISPD: International Society for Peritoneal Dialysis
OR: Odds ratio
95%CI: 95% Confidence interval
SMAP: Stepwise initiation of peritoneal dialysis using Moncrief and Popovich technique
SPIED: Short-term PD Induction and Education technique
